# Long‐term effects of artificial nighttime lighting and trophic complexity on plant biomass and foliar carbon and nitrogen in a grassland community

**DOI:** 10.1002/ece3.9157

**Published:** 2022-08-04

**Authors:** Vinka Anic, Kevin J. Gaston, Thomas W. Davies, Jonathan Bennie

**Affiliations:** ^1^ Environment and Sustainability Institute University of Exeter Cornwall UK; ^2^ School of Biological and Marine Sciences University of Plymouth Plymouth UK

**Keywords:** artificial nighttime lighting, biomass allocation, foliar nitrogen, grassland communities, invertebrate herbivores, plant biomass

## Abstract

The introduction of artificial nighttime lighting due to human settlements and transport networks is increasingly altering the timing, intensity, and spectra of natural light regimes worldwide. Much of the research on the impacts of nighttime light pollution on organisms has focused on animal species. Little is known about the impacts of daylength extension due to outdoor lighting technologies on wild plant communities, despite the fact that plant growth and development are under photoperiodic control. In a five‐year field experiment, artificial ecosystems (“mesocosms”) of grassland communities both alone or in combination with invertebrate herbivores and predators were exposed to light treatments that simulated street lighting technologies (low‐pressure sodium, and light‐emitting diode [LED]‐based white lighting), at ground‐level illuminance. Most of the plant species in the mesocosms did not exhibit changes in biomass accumulation after 5 years of exposure to the light treatments. However, the white LED treatment had a significant negative effect on biomass production in the herbaceous species *Lotus pedunculatus*. Likewise, the interaction between the white LED treatment and the presence of herbivores significantly reduced the mean shoot/root ratio of the grass species *Holcus lanatus*. Artificial nighttime lighting had no effect on the foliar carbon or nitrogen in most of the grassland species. Nevertheless, the white LED treatment significantly increased the leaf nitrogen content in *Lotus corniculatus* in the presence of herbivores. Long‐term exposure to artificial light at night had no general effects on plant biomass responses in experimental grassland communities. However, species‐specific and negative effects of cool white LED lighting at ground‐level illuminance on biomass production and allocation in mixed plant communities are suggested by our findings. Further studies on the impacts of light pollution on biomass accumulation in plant communities are required as these effects could be mediated by different factors, including herbivory, competition, and soil nutrient availability.

## Graphical abstract

Artificial nighttime lighting is increasingly altering the timing and spectra of natural light regimes worldwide. Through a five‐year field experiment, we examined the effects of night‐time artificial illumination (white vs amber light) on plant biomass and foliar nitrogen in grassland mesocosm communities both alone or in combination with invertebrate herbivores. Species‐specific and negative effects of white LED lighting (simulating street lighting at ground‐level illuminance) on plant biomass and shoot/root ratio were found. The activity of herbivores might also be affected by this type of lighting as suggested by the findings of our long‐term study.

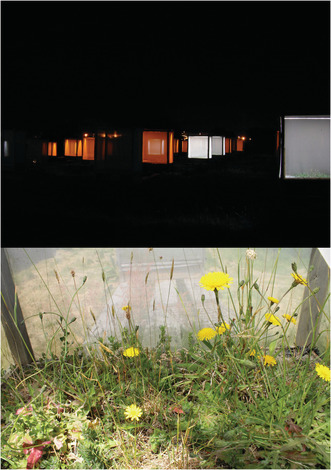

## 1 INTRODUCTION

Natural daily light cycles are increasingly being eroded across the globe as a consequence of the introduction of artificial nighttime lighting. Associated with human settlement, transport networks and industry, these emissions alter the timing, intensity and spectra of natural light regimes (Gaston et al., 2013, 2014). Their effects are such that nearly a quarter of the global land area is already estimated to lie under artificially light polluted nighttime skies (Falchi et al., 2016). The area experiencing direct emissions from artificial light sources is, probably conservatively, estimated currently to be expanding at more than 2% per annum, with localities that are already lit brightening further at a similar rate (Kyba et al., 2017).

Artificial light at night is predicted to constitute a significant anthropogenic pressure on natural biological systems because (i) such systems are organized by daily and seasonal cycles of light and dark (Bradshaw & Holzapfel, 2010; Kronfeld‐Schor & Dayan, 2003); and (ii) there have been no natural analogs, at any timescale, to the nature, extent, distribution, timing or rate of spread of artificial lighting (Gaston et al., 2015). Indeed, a myriad of biological impacts on animal species has been documented (Sanders et al., 2021), including on their physiology (e.g., Dominoni et al., 2013; Grenis & Murphy, 2019), behavior (Baker, 1990; Fullard, 2001; Raap et al., 2015), competitive interactions (Case et al., 1994), mortality (Bukalev et al., 2013), and their abundance and distribution (Davies et al., 2012; Sanders et al., 2015).

By contrast, studies of the impacts of outdoor artificial nighttime lighting on plant species have been surprisingly limited (Bennie et al., 2016). This is despite the fact that some of the earliest reports of the impacts of street lighting concerned the delayed retention of leaves on trees (e.g., Matzke, 1936) and that artificial nighttime lighting is widely used indoors (e.g., in greenhouses) to change timings of growth and flowering of some horticultural crops (e.g., Craig & Runkle, 2013). Nonetheless, it is clear that wild plant species growing in the vicinity of streetlights will experience rather different light regimes than those well away from such light sources (Bennie et al., 2016). This could have important effects on plant growth and development by altering perceived daylength, given that most of the developmental transitions throughout the plant life cycle, such as germination and flowering, are under photoperiodic control (Galvão & Fankhauser, 2015; Lagercrantz, 2009). These photoperiodic responses rely on the activity of photoreceptors, including phytochromes and cryptochromes, which can sense specific wavelengths of light as well as respond to changes in light duration and intensity (Exner et al., 2010; Galvão & Fankhauser, 2015; Smith, 1982).

The available evidence on the effects of photoperiod extension on plant biomass, a key response to understand, comes mainly from low‐irradiance daylength extension treatments (illuminance value around 300–1000 lx) that use incandescent lamps (Adams & Langton, 2005; Hay, 1990), having different spectral characteristics compared with the light sources most commonly used for street lighting. These low‐intensity photoperiod extension treatments also have light levels higher than the illuminance of street lighting at ground level (10–40 lx; Gaston et al., 2014). An increase in dry weight (mostly shoot biomass) has been found to be promoted by these low‐irradiance daylength extension treatments in several grass species at individual level (Hay, 1990; Hay & Heide, 1983; Heide et al., 1985; Solhaug, 1991a, 1991b). A promotive effect of low‐intensity photoperiod extension on biomass acquisition has also been recorded in plants of the grass species *Dactylis glomerata* grown close together (Ryle, 1966). The stimulation of dry matter production due to daylength extension at light intensities much lower than sunlight irradiance (1000–103,000 lx; Gaston et al., 2013) has been mostly related to a positive effect of photoperiod extension on leaf expansion (mainly on leaf length) and plant leafiness (leaf area per unit of plant dry weight; Adams & Langton, 2005; Heide et al., 1985; Solhaug, 1991a, 1991b; Wu et al., 2004).

More directly, high‐pressure sodium (HPS) street lights, which mainly emit yellow and red light, can delay leaf senescence (Massetti, 2018), enhance the size of maize plants (Sinnadurai, 1981), and promote continuous growth in some tree species, as well as flowering in some long‐day plants grown alone at a light intensity of approximately 10 lx (Cathey & Campbell, 1975). Similarly, the presence of the invasive grass species *Bromus tectorum* has been positively associated with nighttime illumination that comes from sodium vapor street lights (Murphy et al., 2021). In contrast, flower density in the leguminous plant *Lotus pedunculatus* has been shown to be reduced by low‐pressure sodium (LPS) street lighting, which emits an almost monochromatic yellow‐orange light (Bennie et al., 2015).

In recent years, LPS and HPS street lamps and other traditional lighting technologies have increasingly been replaced by light‐emitting diode (LED)‐based lamps, typically with a broader “white” light spectrum, including cool white LED street lighting (Bennie et al., 2016). This outdoor lighting technology has a primary peak in the blue portion of the visible spectrum and a wide secondary peak including red light (Elvidge et al., 2010; Gaston et al., 2014). Bennie, Davies, Cruse, Bell, et al. (2018) demonstrated that cool white LED lighting at ground‐level illuminance of approximately 30 lx, as well as a light treatment simulating LPS lighting (approx. 18 lx) had a positive effect on aboveground biomass in the grass species *Holcus lanatus* growing in a grassland community. An increase in basal stem diameter under neutral white LED lighting (approx. 50 lx), which has different percentages in the blue and red bands of the light spectrum compared with cool white LEDs, has been found in the wildflower species *Asclepias syriaca*, the promotive effect of the lighting treatment on plant growth being greater in single plants than in conspecifics grown close together (Hey et al., 2020).

These positive plant responses to photoperiod extension with low light intensity suggest that the low light levels of the current street lighting technologies at ground level might promote dry matter production in many species. However, individual photoperiodic responses to artificial nighttime lighting could be affected by interspecific interactions, such as competition for limiting resources, including soil nitrogen and water. The availability of these resources can influence both productivity and composition of plant communities (Craine & Dybzinski, 2013; Laughlin & Abella, 2007). Plant growth and development are greatly dependent on soil nitrogen availability as timing of flowering as well as leaf photosynthetic capacity rely on nitrogen concentration (Evans, 1989; Lin & Tsay, 2017). Most of the foliar nitrogen is invested in the proteins responsible for photosynthesis (e.g., Rubisco; Evans, 1989; Hikosaka, 2010). Consequently, belowground competition for nitrogen has been described as one of the most important drivers of changes in plant community structure in unfertilized environments (Wilson & Tilman, 1993). Some plant species are able to pre‐empt nutrient supplies from coming into contact with other species by allocating more biomass to roots (Fargione & Tilman, 2006). Similarly, taller species can pre‐empt light supplies from smaller neighbors via shade (Tow & Lazenby, 2001), which in turn may reduce plant biomass due to the effect of light limitation on photosynthesis (Craine & Dybzinski, 2013; Ringselle et al., 2017). Insect herbivory can also decrease biomass in some species in plant communities, as demonstrated by experiments where insect herbivores have been excluded (Bonser & Reader, 1995; Carson & Root, 1999).

An increase in the total biomass production of pots with alien and native plants grown close together has been found under all‐night exposure to fluorescent lamps (approx. 28 lx) (Speißer et al., 2021), which have a different spectral composition compared with cool white LED lighting. In contrast, a study conducted by Bennie, Davies, Cruse, Inger, et al. (2018) recorded a negative effect of nighttime illumination with cool white LED lighting at a light level of approximately 10 lx on both the cover of total leguminous forbs and the flowering of the leguminous plant *L. pedunculatus* in experimental grassland communities. The white LED treatment also significantly increased the foliar carbon to nitrogen ratio of *L. pedunculatus*, this plant response being only evaluated in this legume in the experimental plant communities (Bennie, Davies, Cruse, Inger, et al., 2018).

What has been lacking to date is an experimental assessment of the impacts of artificial nighttime lighting and trophic complexity on plant biomass and foliar carbon and nitrogen in a community (rather than single species) context, at light levels and spectra likely to be encountered in the field. Here, we report the results of a five‐year experiment using the same plant communities that were previously studied by Bennie, Davies, Cruse, Inger, et al. (2018). Grassland mesocosm communities, alone or in combination with invertebrate herbivores and predators, were exposed to light treatments that simulated the spectral distribution of emissions from LPS lamps and cool white LED street lighting at ground‐level illuminance under field conditions. In these grassland communities, plant biomass production and allocation responses of individual species to the light treatments were evaluated.

## 2 MATERIALS AND METHODS

### 2.1 Experimental design

The field experiment ran from 2012 to 2016 and was designed to investigate the effects of different sources of artificial nighttime lighting on artificial ecosystems (“mesocosms”) of grassland plants growing alone or in combination with invertebrate herbivores and predators (Bennie et al., 2015).

**FIGURE 1:**
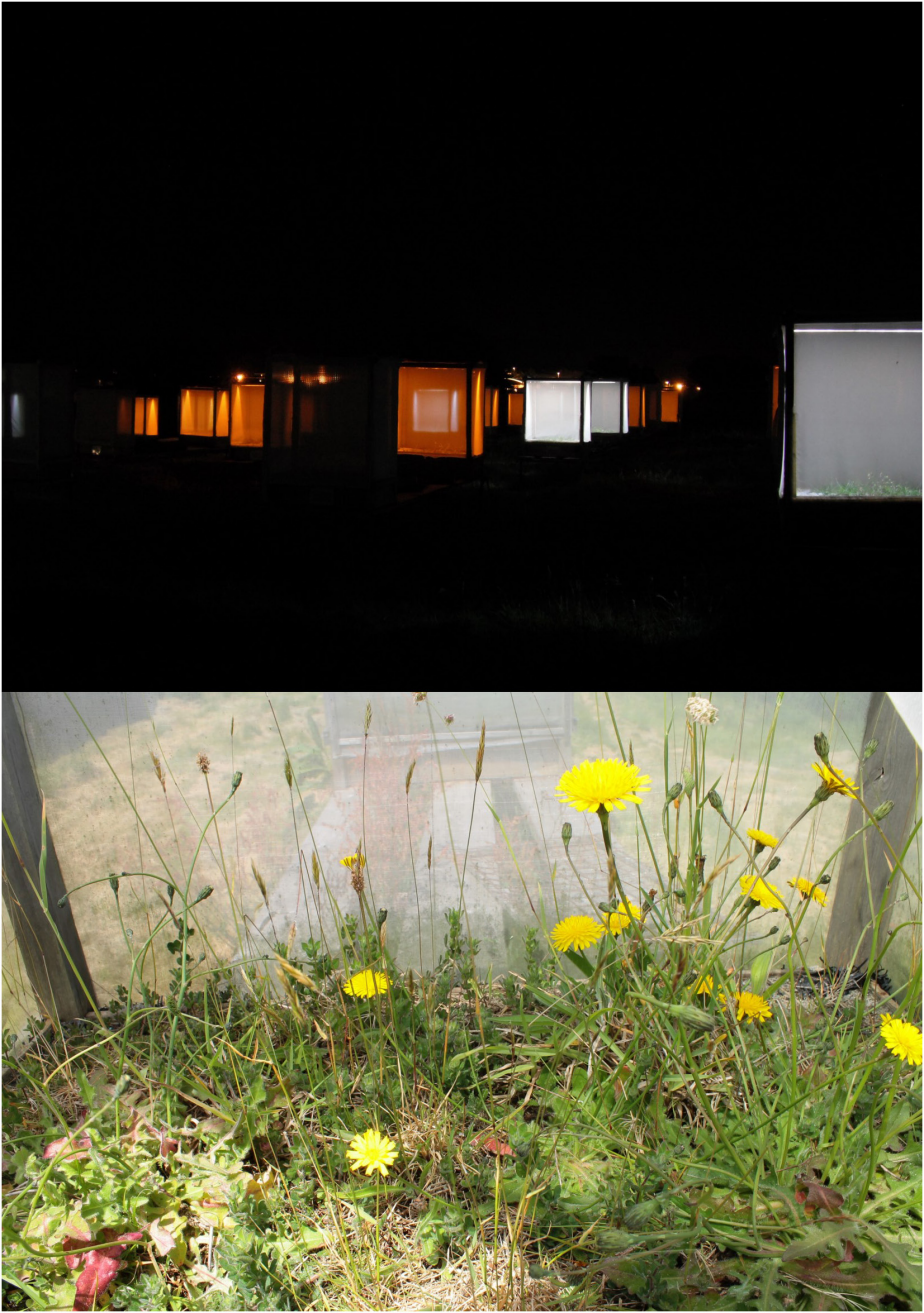
Top: nighttime view of the experiment, July 2012. Photo: James Duffy. Bottom: example plant community, June 2015

We established 54 experimental grassland “mesocosms” outdoors in July 2012 (Figure 1 and Figure S1) at the University of Exeter's Penryn Campus (50°10’ N, 5°7’ W, altitude 106 m). Each mesocosm consisted of a 1 m × 0.5 m × 0.2 m trough, lined with woven plastic textile for drainage and filled with coarse builder's sand, and mounted on a wooden platform 0.75 m above the ground. In each mesocosm, 72 individual grassland plants were planted, representing four individuals of each of 18 common European grassland species grown in spring from seeds gathered locally from wild plants in 2011. These were selected to be representative of species common in roadside verges in the local area and comprised six grasses (*Anthoxanthum odoratum, Agrostis tenuis, Holcus lanatus, Cynosurus cristatus, Dactylis glomerata*, and *Festuca ovina*), four legumes (*Lotus corniculatus, Lotus pedunculatus, Trifolium dubium*, and *Trifolium pratense*), and eight non‐leguminous forbs (*Leucanthemum vulgare, Achillea millefolium, Leontodon saxatilis, Hypochaeris radicata, Prunella vulgaris, Centaurea nigra, Ranunculus acris*, and *Plantago lanceolata*). These species could be influenced by sources of street lighting. Seedlings were transplanted in a randomized grid pattern 5 cm apart within the central section of each mesocosm in July 2012. A standard nutrient solution was applied to each mesocosm during July 2012 to establish initial plant growth. The plants in the experimental communities were not grown under continuous nutrient supply, as we wanted to simulate a natural environment as experienced by grassland plants. Grasslands have generally been described as low‐nutrient environments (Mamolos et al., 1995). Some plant species are expected to be dominant and others rarer at the end of the experiment. This would make our plant communities more similar to wild grassland communities which naturally have rarer species. The invertebrate community was isolated in each mesocosm by a wooden frame 1 m tall and lined with fine anti‐thrip mesh, with a zip for access for maintenance and measurements.

Three levels of trophic complexity were established in the mesocosms. Eighteen mesocosms contained *plants only*, 18 (bitrophic treatments) contained *both plants and herbivores*, and 18 (tritrophic treatments) contained *plants, herbivores, and predators*. All mesocosms were treated with biodegradable insecticide (pyrethrin) and molluscicide (ferric phosphate pellets) in 2012, 1 year prior to the introduction of the invertebrates. The plants‐only treatment mesocosms contained the grassland plant species and were treated at regular intervals with the biodegradable insecticide and ferric phosphate pellets to prevent the establishment of invertebrate populations from the surrounding environment. Both the bitrophic and tritrophic treatments received phased introductions of 20 individuals of the pea aphid *Acyrthosiphon pisum* and 30 individuals of the gray field slug *Deroceras reticulatum* from May to June 2013 as described by Bennie, Davies, Cruse, Inger, et al. (2018). Populations of *A. pisum* persisted throughout the experiment, peaking during summer each year and overwintering as both eggs and adults; populations of *D. reticulatum* persisted until autumn/winter 2014 when numbers declined drastically in all mesocosms. A further 30 individuals were introduced from June to September 2015. *A. pisum* is a specialist feeder on legumes, and individuals were gathered from wild populations feeding on the leguminous forb *Lotus pedunculatus*. The density of *A. pisum* has been described to range from approximately 5 to 116 individuals per m^2^ in herbaceous vegetation (Obrycki et al., 1997). *Deroceras reticulatum* is a generalist omnivore, feeding predominantly on the foliage of a wide range of plant species. In addition, the tritrophic treatments received introductions of adult individuals of the predatory ground beetle *Pterostichus melanarius* and of the ladybird *Adalia bipunctata* (Bennie, Davies, Cruse, Inger, et al., 2018). *A. bipunctata* is a specialist predator of aphids; *P. melanarius* is a generalist predator that will feed on the most available prey, including both slugs and aphids (Pollet & Desender, 1987). This predator locates its prey primarily through visual cues (visual predator).

Three light treatments, white light‐emitting diode (white LED; simulating cool white LED street lighting), monochromatic amber LED (simulating low‐pressure sodium street lighting), and control (unlit) were allocated to mesocosms in a cross‐factorial design with trophic level, with six replicates of each light and trophic treatment combination (nine possible light/trophic combinations) randomly distributed within a grid pattern (Figure S1). The non‐control light treatments (white and amber light) each consisted of a strip of LEDs mounted on a wooden bar across the top of the mesocosm and facing downwards. The white LED treatment consisted of “cool white” LEDs, with a spectrum similar to those in commercial LED street lighting systems (Figure S2). The amber light treatment consisted of a virtually monochromatic LED strip with a single narrow peak in the orange portion of the spectrum, at around 588 nm, aiming to simulate the peak emittance of monochromatic low‐pressure sodium (LPS) lighting at 589.3 nm (Figure S2). Both lighting treatments provided an illuminance of approximately 10 lx at the unshaded sand surface and 15 lx at 20 cm height. These illuminance levels are within the range of those typical of roadside vegetation under street lights (Bennie et al., 2016). Unlit “control” treatments reproduced the mounting bar of the lit treatments but had no light source. Light treatments were switched on at sunset (when ambient light levels fell below 70 lx) and off at sunrise (above 110 lx).

### 2.2 Sampling for plant biomass and foliar carbon and nitrogen

At the end of the experiment (July 2016), leaf samples of all plant species surviving in each mesocosm were taken and sent for elemental and isotopic analysis of carbon (C) and nitrogen (N). Samples were taken from six leaves from three separate plants, and then mixed together, except in cases where there were less than three plants present, in which case six leaves were taken from as many plants as were present. The aim was to study both the availability of nitrogen for leaf photosynthesis and the partitioning of foliar carbon and nitrogen under the light treatments. Following the end of the experiment (August 2016), the mesocosms were fully dismantled and all plant biomass was retained, washed free of soil and sand, sorted by species, and air dried in mesh bags in a drying room at a constant temperature. Above‐and belowground biomass were separated and weighed.

### 2.3 Data analyses

Treatment effects on plant biomass and foliar nitrogen and carbon datasets for each study species and plant species richness and total plant biomass per mesocosm were analyzed using general linear models (GLMs) in the R statistical software (v. 3.5.1; R Core Team 2018). Lighting treatment (unlit controls, LPS‐amber light, and white LED) and trophic treatment (presence or absence of herbivores and presence or absence of predators) were included as fixed factors. Model quality was assessed by Akaike information criterion (AIC) values. To do so, models consisting of full combinations of fixed factors and interaction terms were tested, and significance values are reported here for the best model for each study species (Supplementary material). The GLMs were checked for over‐dispersion by inspecting both residual deviance and residual degrees of freedom. Normality of residuals and homogeneity of variance were checked visually using normal Q‐Q plots, and scale‐location plots, respectively. Post hoc pairwise comparisons with a *p*‐value adjustment equivalent to the Tukey HSD test were conducted using the emmeans package in R (Lenth et al., 2019).

## 3 RESULTS

### 3.1 Biomass accumulation and partitioning

Of the 18 study species, five species (*Cynosurus cristatus, Leucanthemum vulgare, Leontodon saxatilis, Ranunculus acris*, and *Trifolium dubium*) had surviving individuals in only a very small number of mesocosms (just one mesocosm per light treatment for two of the species) by the end of the experiment (Table S1). Due to this, total plant biomass (aboveground + belowground) was analyzed for the remaining 13 study species (Figures 3 and 4), all of which had three or more mesocosms with surviving individuals for each light and trophic treatment combination (Table S1).

**FIGURE 2:**
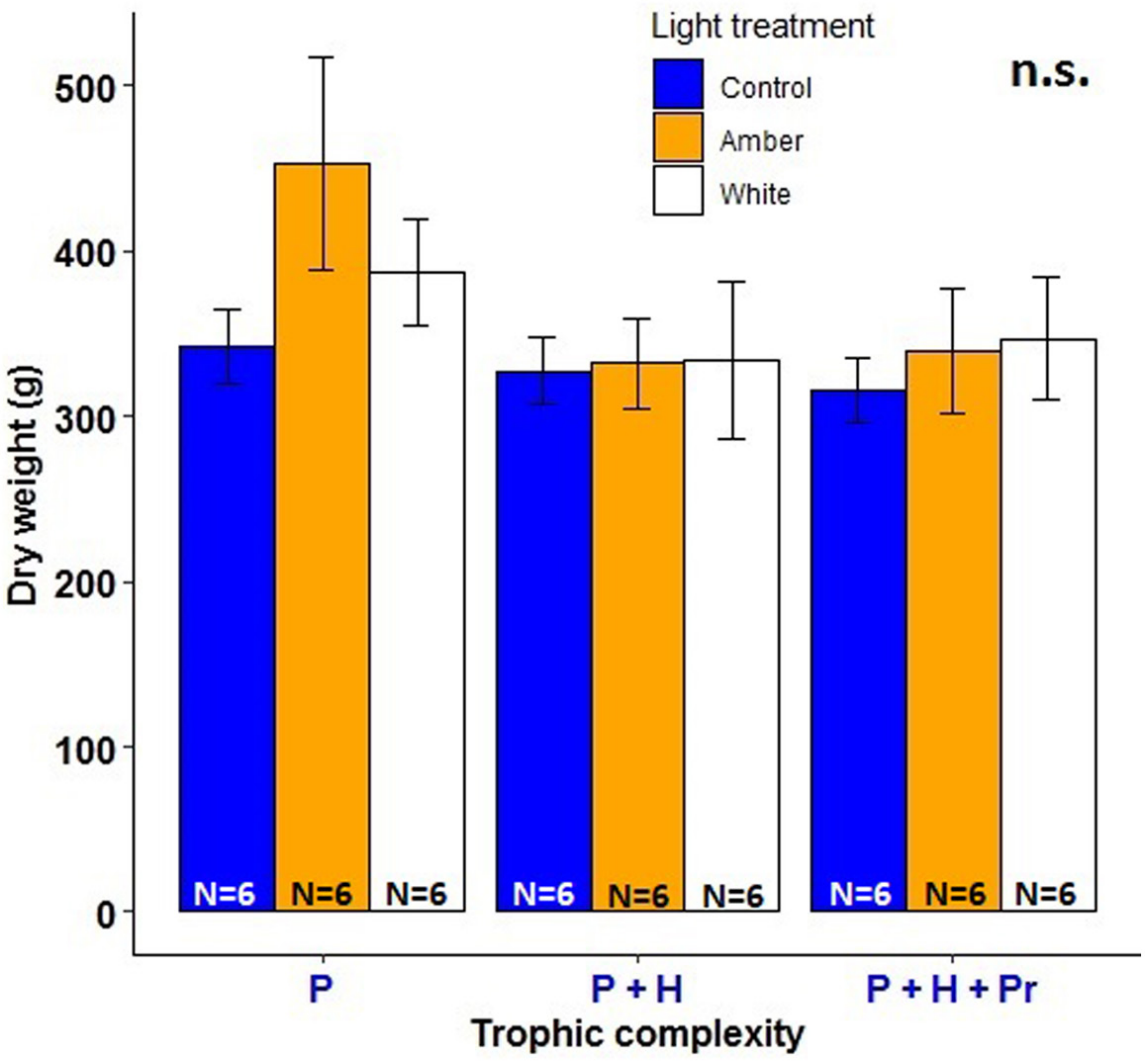
Total plant biomass (g/0.5 m × 0.2 m) per mesocosm. Error bars represent SEM. Experimental treatments: C = unlit control, A = amber light (simulating LPS lighting), and W = white LED lighting. Trophic complexity: P = plants, P + H = plants and herbivores, and P + H + Pr = plants, herbivores, and predators. n.s. indicates no statistically significant differences. N = sample size.

**FIGURE 3:**
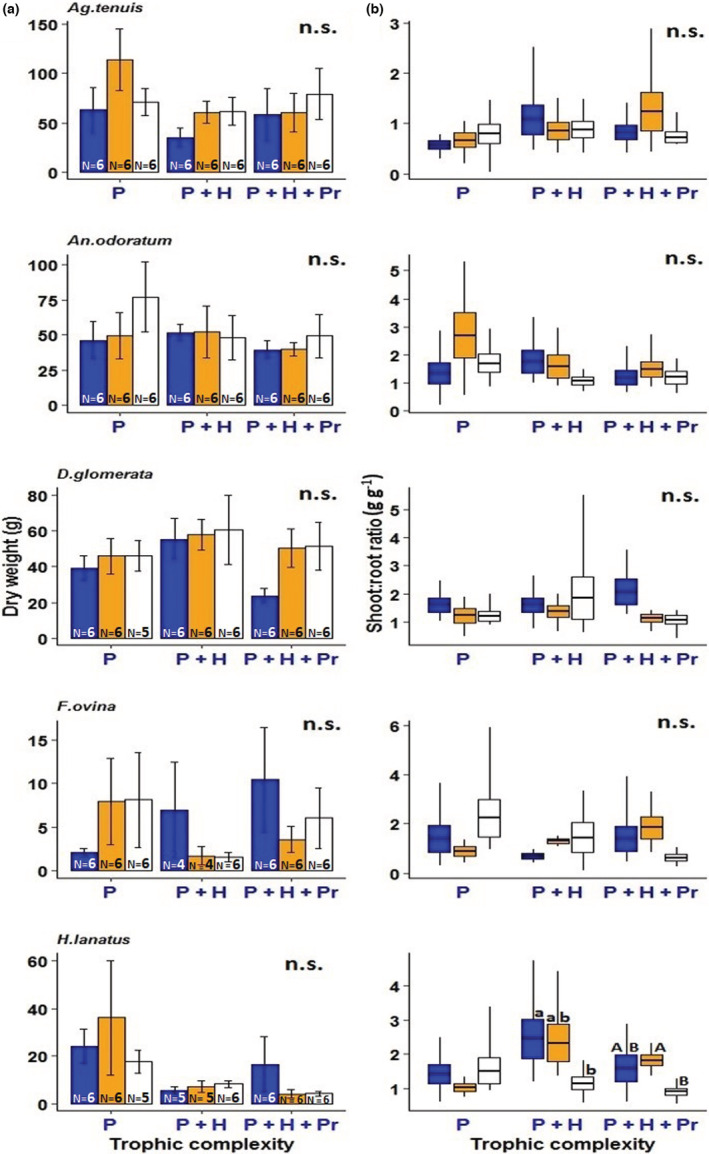
(a) Biomass production, and (b) shoot/root ratio of five grass species. Experimental treatments: C = unlit control, A = amber light (simulating LPS lighting), and W = white LED lighting. Trophic complexity: P = plants, P + H = plants and herbivores, and P + H + Pr = plants, herbivores, and predators. Error bars represent SEM. Box plots (B) show median values (thick line), SEM (colored bar), and minimum/maximum values. Different letters indicate statistically significant differences between means at *p* < .05., and n.s. indicates no statistically significant differences. N = sample size.

**FIGURE 4:**
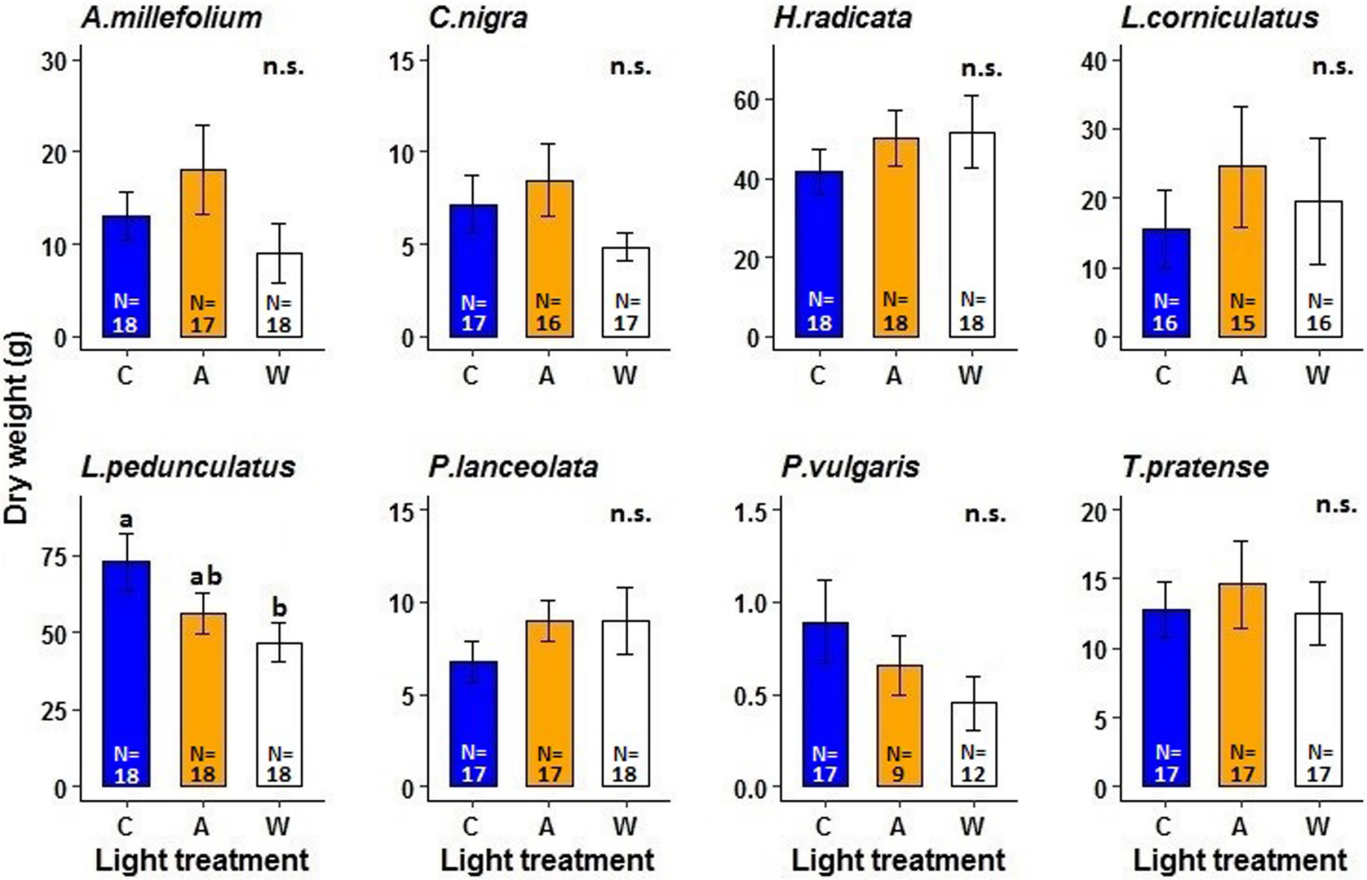
(A) Biomass production of eight herbaceous species. Experimental treatments: C = unlit control, A = amber light (simulating LPS lighting), and W = white LED lighting. Different letters above error bars (SEM) indicate statistically significant differences between means at *p* < .05. n.s. indicates no statistically significant differences. N = sample size.

Total plant biomass per mesocosm (including all plant species surviving in each mesocosm; maximum number of species = 17) and plant species richness were unaffected by the light treatments simulating street lighting at ground‐level illuminance (Figure 2, Tables S2 and S5). Likewise, plant biomass was not significantly affected by the amber light treatment in any of the 13 grassland species (Figures 3 and 4, Table S3). In contrast, the white LED treatment significantly decreased biomass production in the herbaceous species *Lotus pedunculatus* compared with the unlit controls (Tukey test, *p* = .0466; Figure 4, Table S3). Similarly, there was a marginally significant reduction in the total biomass of the herbaceous species *Prunella vulgaris* under the white LED treatment (Tukey test, *p* = .084; Figure 4, Table S3). Shoot: root ratios of most of the study species did not differ between the lighting treatments (Figure 3b, Table S3). However, this ratio was significantly lower for the grass species *Holcus lanatus* under the white LED treatment compared with the controls, but only in the presence of invertebrate herbivores (Tukey test, *p* = .027; Figure 3b, Table S3). For this species, shoot: root ratio was also significantly lower under white LED lighting compared with the treatment simulating LPS lighting (Tukey test, *p* = .042; Figure 3b), but only in the presence of both herbivores and predators. The trophic treatment had no significant effect on total plant biomass per experimental grassland community (Table S2).

### 3.2 Foliar carbon and nitrogen

The percentages of foliar nitrogen (N) and foliar carbon (C) per unit dry weight were determined for 10 of the 18 study species, which had three or more mesocosms per each light and trophic treatment combination, each with sufficient leaf biomass to sample after 5 years of exposure to the treatments.

**FIGURE 5:**
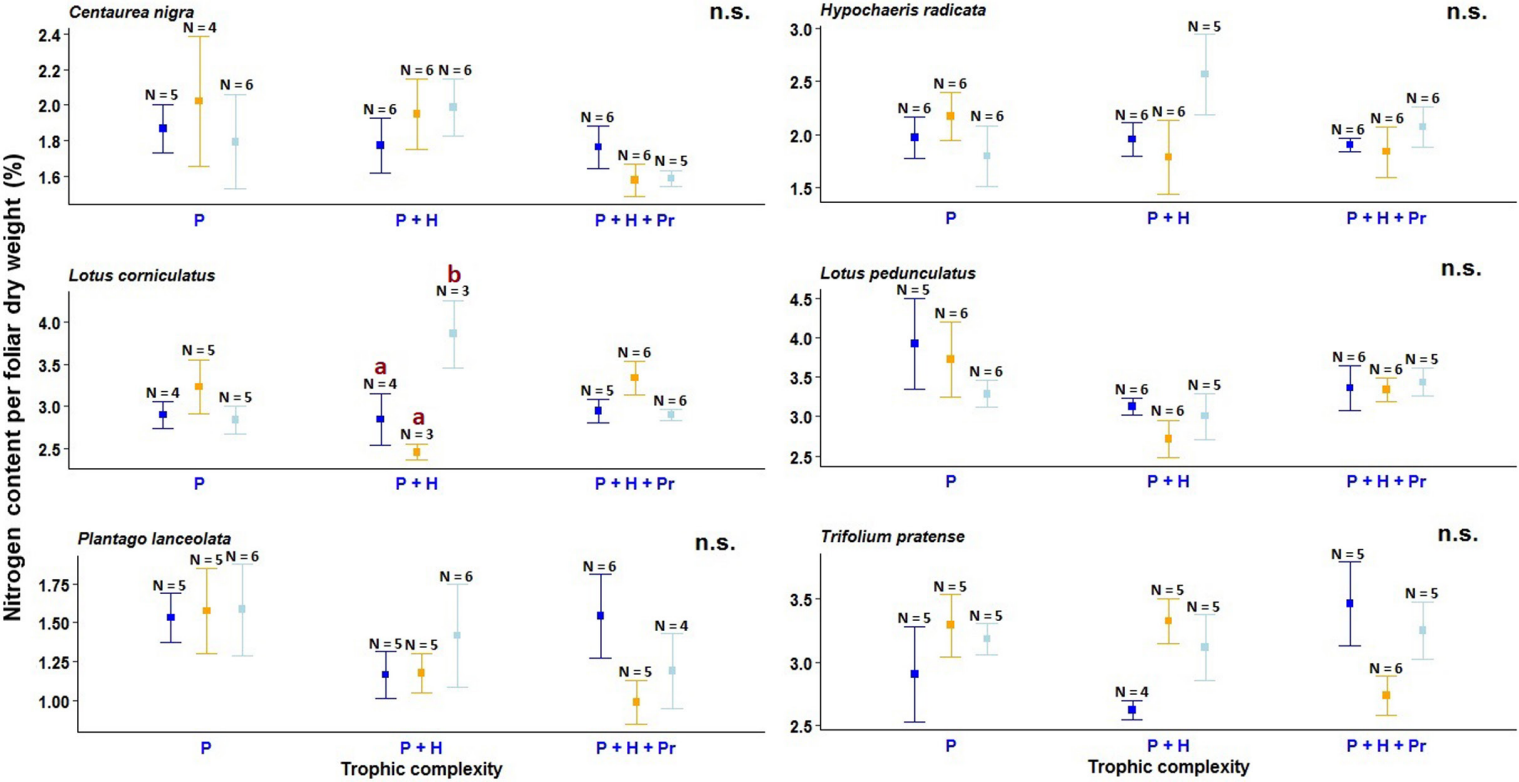
Leaf nitrogen content (%) of six herbaceous species. Experimental treatments: Blue = unlit control, orange = amber light (simulating LPS lighting), and light blue = white LED lighting. Trophic complexity: P = plants, P + H = plants and herbivores, and P + H + Pr = plants, herbivores, and predators. Different letters above error bars (SEM) indicate statistically significant differences between means at *p* < 0.05. n.s. indicates no statistically significant differences. N = sample size.

Leaf nitrogen content (%) was not affected by any of the lighting treatments in most of the study species (Figures 5 and Tables S3 and S4). The white LED treatment significantly increased leaf nitrogen content in *Lotus corniculatus* compared with the controls, but only in the presence of herbivores (Tukey test, *p* = .025; Figure 5). As a result, leaf C:N ratio was significantly lower in plants of this species that were exposed to the white LED treatment in the presence of invertebrate herbivores, compared with the unlit controls (Tukey test, *p* = .025). Leaf C:N ratio did not vary significantly among the light treatments in the other study species (Figure S3, Table S4).

## 4 DISCUSSION

Most of the study species in the experimental grassland communities did not exhibit changes in biomass accumulation and allocation patterns after 5 years of exposure to photoperiod extension simulating street lighting at ground‐level illuminance (LPS and cool white LED lighting). Artificial nighttime lighting also had no effect on foliar carbon or nitrogen in most of the species. However, some species did show individual effects, suggesting that in mixed plant communities such effects may be species‐specific. Moreover, plant responses to artificial nighttime lighting could be dependent on the spectral distribution of light as the changes in both plant biomass and leaf N content were only recorded under cool white LED lighting. Previous work carried out by Bennie, Davies, Cruse, Inger et al. (2018) on the same experimental plant communities also found a significant effect of this type of lighting (4 years of exposure) on plant responses including number of inflorescences and leaf C:N ratio, which were only studied in *Lotus pedunculatus*. Both plant responses in this species were decreased by cool white LED lighting compared with unlit controls after 4 years of exposure to the light treatment. This suggests that the negative effect of artificial nighttime lighting on foliar C/N ratio in *L. pedunculatus* might be due to changes in resource allocation between leaves and flowers. However, the leaf C/N ratio of *L. pedunculatus* was unaffected by the white LED treatment in the present research. Hence, further research is required to determine the explanatory mechanism behind our findings.

Unlike the common promotive effect of low‐irradiance photoperiod extension treatments with incandescent lamps on plant biomass in individual species in isolation, the light treatment simulating LED‐based white street lighting had a negative effect on biomass production in the herbaceous species *L. pedunculatus* in the experimental plant communities. Likewise, a marginally significant decrease in biomass was found in the herbaceous species *Prunella vulgaris* under cool white LED lighting. These findings together with previously recorded reductions in both the inflorescence abundance of *L. pedunculatus* and total legume cover in the grassland communities under the white LED treatment (Bennie, Davies, Cruse, Inger et al., 2018) suggest that competitive interactions in the plant communities might account for the negative response of plant biomass of *L. pedunculatus* to cool white LED lighting. However, competition for light would not account for the decrease in biomass recorded in *L. pedunculatus* under white LED lighting as suggested by the absence of a significant increase in shoot biomass in the plant communities exposed to this lighting treatment. Conversely, the reduction in biomass in *L. pedunculatus* under artificial nighttime lighting might be explained by an indirect positive effect of cool white LED lighting on plant competition for limiting nutrients. Through a promotive effect on the activity of plant photoreceptors, the white LED treatment could have stimulated plant growth of some species in winter, when most plant species are dormant. Supplemental lighting that comes from light sources that emit red light (e.g., HPS lighting) has been shown to promote plant growth in winter (Cathey & Campbell, 1979; Grimstad, 1987). This suggests that cool white LED lighting might have increased plant competition for nutrients through a higher nutrient uptake required to sustain continuous plant growth (e.g., growth in the cold season). However, further studies on the direct effects of the white LED treatment on plant growth in single species at different soil nutrient levels are required to determine the mechanisms behind the reduction in dry weight of *L. pedunculatus* under white LED lighting at ground‐level illuminance in the grassland communities.

The percentages of foliar nitrogen determined for most of the study species were low (mean nitrogen content <2%) compared with nitrogen content in leaves described for several herbaceous species (e.g., Cornelissen & Thompson, 1997). Some species might have reached a baseline leaf nitrogen concentration, as indicated by leaf nitrogen percentages lower than 1.5% determined after 5 years of exposure to the light treatments (Figures 5, Figure S3). This suggests that soil nitrogen availability might have been a limiting factor for plant biomass accumulation in some of the study species under artificial nighttime lighting, as foliar nitrogen has been positively related to inorganic N availability (Goedhart et al., 2010). This is in accordance with the availability of soil nutrients in grasslands, most of which are low‐nutrient environments (Mamolos et al., 1995). In contrast, most of the studies on the effects of low‐irradiance daylength extension (that comes from incandescent or fluorescent lamps) on plant biomass have been conducted under continuous nutrient supply (e.g., Heide et al., 1985; Ryle, 1966), which highlights the role of nutrient availability on the positive responses of dry matter production to photoperiod extension.

The interaction between the white LED treatment and the presence of herbivores significantly reduced the mean shoot/root ratio of the grass species *Holcus lanatus* compared with the unlit controls with herbivores. This suggests that the activity of some herbivores, specifically mollusks that feed on this grass species, may have been promoted by cool white LED lighting, which in turn could have decreased aboveground biomass in *H. lanatus*. However, previous research conducted in the mesocosms of the present study has determined that this lighting treatment has no effect on the abundance of a generalist herbivore mollusk in the absence of a visual predator (Bennie, Davies, Cruse, Inger, et al., 2018). This suggests that white LED lighting might have reduced shoot biomass in *H. lanatus* by promoting the foraging activity of herbivores rather than increasing their abundance.

The white LED treatment also increased leaf N percentage in the legume *Lotus corniculatus*, but only in the presence of invertebrate herbivores. Further investigation on herbivore damage under white LED lighting is required to determine the explanatory mechanism behind this finding.

No general effects of long‐term exposure to artificial light at night, specifically cool white LED lighting at ground‐level illuminance, on plant biomass responses were found in our study, suggesting that this type of lighting would not account for prominent shifts in the structure of grassland communities. In addition, no changes in plant species richness in communities exposed to the white LED treatment were recorded. However, plant biomass responses to artificial nighttime lighting in mixed communities could be both species‐specific and negative as predicted by the findings of our long‐term study. Direct impacts of cool white LED lighting on plant herbivory are also suggested by the present research, as the interaction of the white LED treatment and the presence of arthropod herbivores had effects on biomass partitioning and foliar nitrogen in some of the study species. Therefore, long‐term plant biomass accumulation and allocation responses to artificial nighttime lighting can be complex as they may be mediated by different factors, including herbivory, competition, and soil nutrient availability.

Plant communities are faced with multiple drivers of change, including global habitat loss, climate change, and nutrient pollution. Even if light pollution may seem to be less of an issue in isolation, it is important to put it in this context of multiple stressors. For instance, nutrient pollution might affect the direction and magnitude of the effects of artificial light at night on biomass production in plant communities as plant growth is dependent on soil nutrients. Further studies on the influence of the current street lighting technologies on plant growth and development in road verge plant communities would be of great importance as these environments sustain high levels of biodiversity (O'farrell & Milton, 2006) and constitute the last refuge for some native species (Perring, 1969).

## AUTHOR CONTRIBUTIONS

**Vinka Anic:** Data curation (equal); formal analysis (lead); investigation (lead); writing – original draft (lead); writing – review and editing (equal). **Kevin J. Gaston:** Conceptualization (lead); funding acquisition (lead); methodology (equal); project administration (equal); resources (lead); supervision (equal); writing – review and editing (equal). **Thomas W. Davies:** Methodology (equal); writing – review and editing (equal). **Jonathan Bennie:** Data curation (equal); methodology (equal); project administration (equal); supervision (equal); writing – review and editing (equal).

## CONFLICT OF INTEREST

The authors have no conflict of interest to declare.

## Supporting information


Data S1

## Data Availability

The datasets presented in this paper are available in the Figshare data repository with the identifier https://doi.org/10.6084/m9.figshare.20304039.
