# Supplementary material for: Long‐term effects of artificial nighttime lighting and trophic complexity on plant biomass and foliar carbon and nitrogen in a grassland community
Source: Ecol Evol. 2022 Aug 4;12(8):e9157. doi: 10.1002/ece3.9157 (PMC9352868; doi:10.1002/ece3.9157)
Supplement: Supplementary file 1 — Data S1 [file ECE3-12-e9157-s001.pdf]

SUPPORTING INFORMATION

Supplementary Figures S1 – S3, Supplementary Tables S1 – S5.

Long-term effects of artificial nighttime lighting and trophic complexity on plant biomass and foliar carbon and nitrogen in a grassland community

Vinka Anic[1]

Kevin J. Gaston[1]

Thomas W. Davies[2]

Jonathan Bennie[1]

[1]Environment and Sustainability Institute, University of Exeter, Cornwall, UK

[2]School of Biological and Marine Sciences, University of Plymouth, UK

Correspondence

Vinka Anic

Environment and Sustainability Institute, University of Exeter, Cornwall, UK

Email: vinkanicthomas@protonmail.com

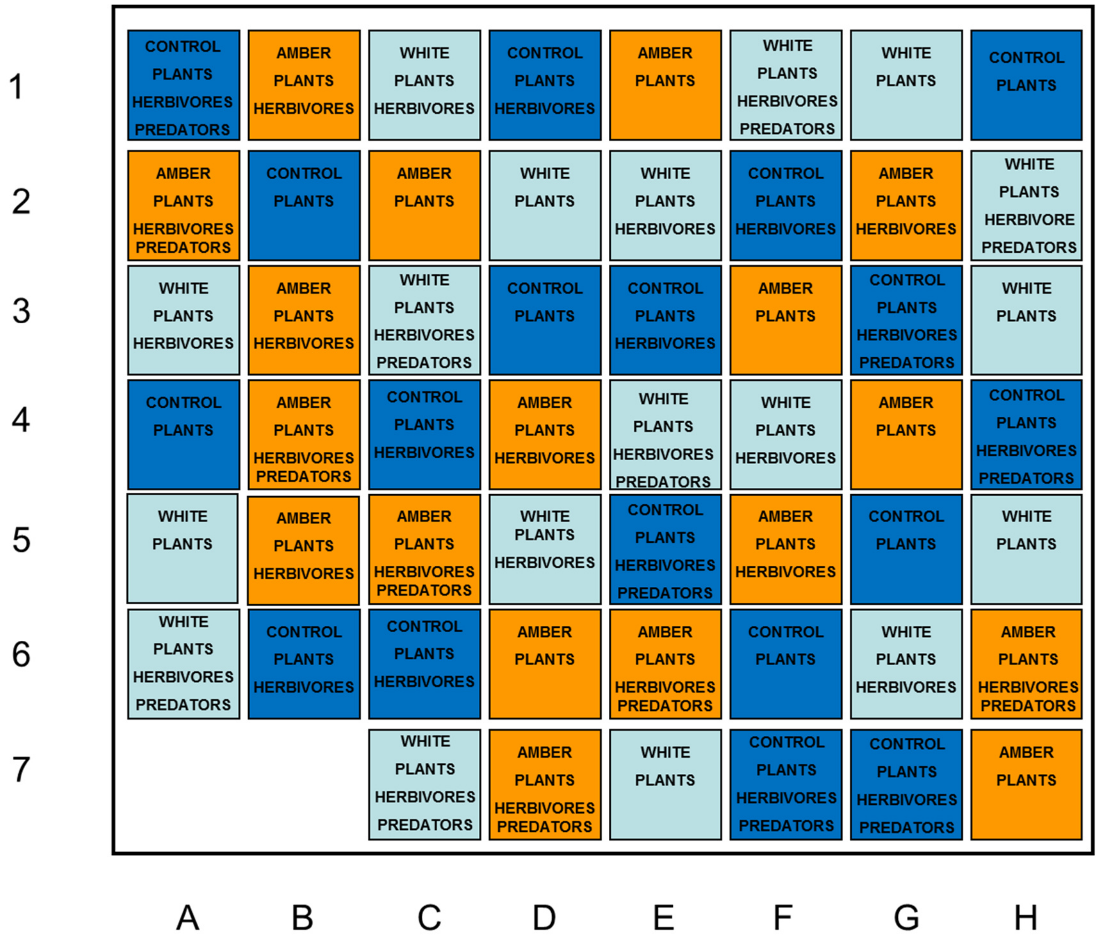

FIGURE S1. Layout of experimental design with 54 mesocosms, and randomly allocated light treatments and levels of trophic complexity.

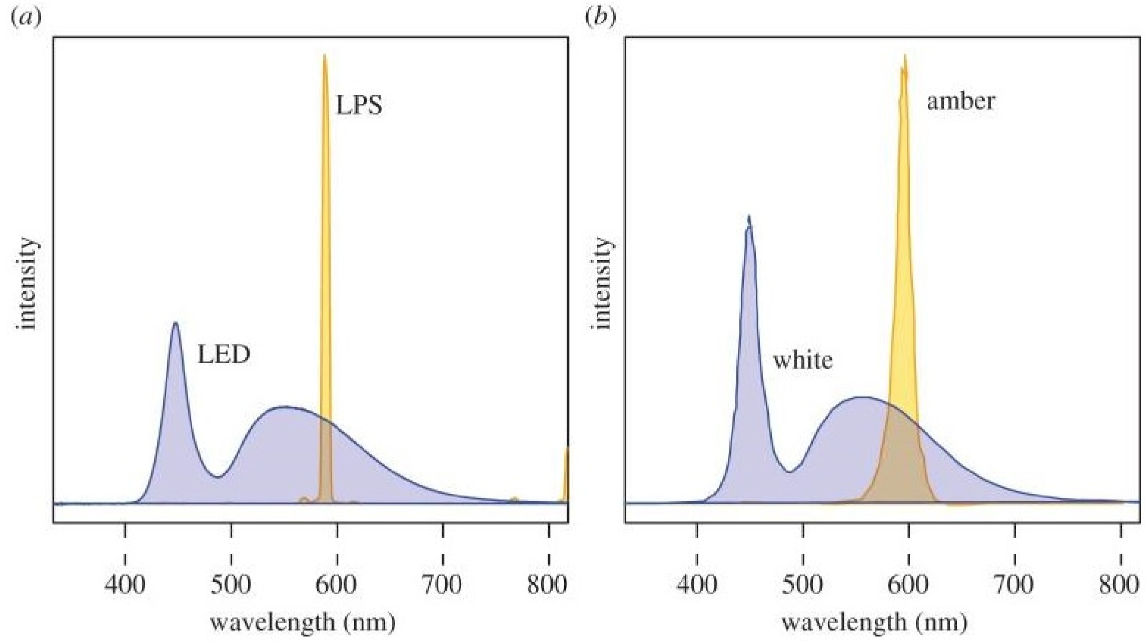

FIGURE S2. (a) Spectral distribution of light from two street light types (LPS lamps and commercial white LED lights) and (b) spectral distribution of light measured in the two experimental light treatments (white and amber LEDs).

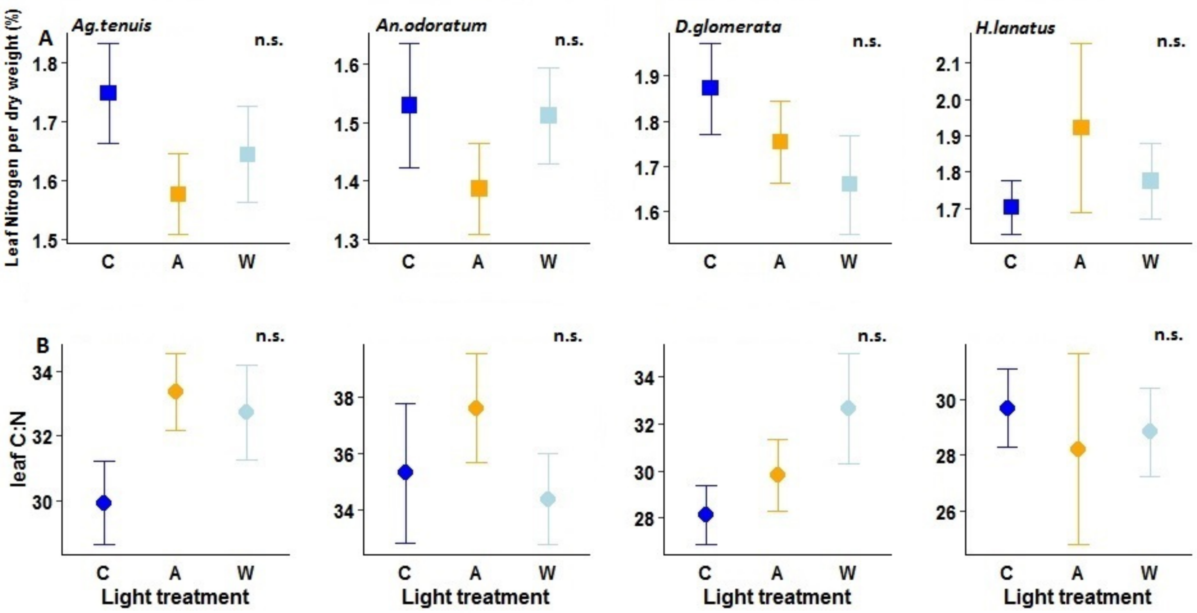

FIGURE S3. (A) Leaf nitrogen content (%), and (B) leaf C:N ratio of four grass species. Experimental treatments: C = unlit control, A = amber light (simulating LPS lighting), and W = cool white LED lighting. Error bars represent SEM. n.s. indicates no statistically significant differences.

TABLE S1. Number of mesocosms with surviving individuals per plant species and per each light and trophic treatment combination. Light treatments: C = unlit control, A = amber light (simulating LPS lighting), and W = white LED lighting. Trophic complexity: T1 = plants, T2= plants and herbivores, and T3= plants, herbivores, and predators. *Sample not included in the GLM for *Prunella vulgaris*.

**Species**	**C**			**A**			**W**			**Total**
**Grasses**	**T1**	**T2**	**T3**	**T1**	**T2**	**T3**	**T1**	**T2**	**T3**	
Agrostis tenuis	6	6	6	6	6	6	6	6	6	54
Anthoxanthum odoratum	6	6	6	6	6	6	6	6	6	54
Cynosurus cristatus	0	0	1	0	1	2	0	0	2	6
Dactylis glomerata	6	6	5	6	6	6	6	6	6	53
Festuca ovina	6	6	6	4	4	6	6	6	6	50
Holcus lanatus	6	6	5	5	5	6	6	6	6	51
**Herbs**										
Achillea millefolium	6	6	6	5	6	6	6	6	6	53
Centaurea nigra	5	6	6	4	6	6	6	6	5	50
Hypochaeris radicata	6	6	6	6	6	6	6	6	6	54
Leontodon saxatilis	1	2	3	4	6	3	4	3	4	30
Leucanthemum vulgare	3	2	0	0	0	2	4	0	2	13
Lotus corniculatus	5	5	6	5	4	6	5	6	5	47
Lotus pedunculatus	6	6	6	6	6	6	6	6	6	54
Plantago lanceolata	6	5	6	5	6	6	6	6	6	52
Prunella vulgaris	5	6	6	1*	6	3	3	4	5	39
Ranunculus acris	3	2	4	0	1	1	0	1	2	14
Trifolium dubium	0	0	2	1	0	0	1	0	0	4
Trifolium pratense	6	6	5	5	6	6	5	6	6	51

TABLE S2. Analysis of deviance for GLM fits. Plant biomass per mesocosm (including 18 study species) was modelled as a function of the light treatment (L1 = unlit control, L2 = amber light (simulating LPS lighting), and L3 = white LED lighting), and trophic treatment (T1 = plants only, T2= plants and herbivores, and T3= plants, herbivores, and predators.). Significance tested against its intercept only model (null model). df = degrees of freedom. Model and error structures are specified. Coefficients relative to the base level of each factor (light treatment = unlit controls; trophic treatment = plants only).

**Fixed effects**	**Estimate**	**Std. error**	**t value**	**P**
**Total biomass per mesocosm**				
glm(formula = Total biomass ~ Light treatment +Trophic treatment, family = Gamma, link = identity)				
Intercept	369.54	27.06	13.658	$<2e^{-16}$
L2	40.11	28.41	1.412	0.1644
L3	25.59	27.79	0.921	0.3617
T2	-58.92	29.50	-1.997	0.0514
T3	-56.79	29.58	-1.920	0.0607
Deviance Null 3.2001 (df = 53) , Residual 2.7292 (df = 49)				

TABLE S3. Analysis of deviance for GLM fits. Plant biomass and shoot/root ratio per species (N = 13) were modelled as a function of light treatment (L1 = unlit control, L2 = amber light (simulating LPS lighting), and L3 = white LED lighting), and trophic treatment (T1 = plants only, T2= plants and herbivores, and T3= plants, herbivores, and predators). Significance tested against its intercept only model (null model). df = degrees of freedom. Model and error structures are specified for each model. Asterisk (*) indicates that an outlier was not included in the analysis. Coefficients relative to the base level of each factor (light treatment = unlit controls; trophic treatment = plants only).

Fixed effects	Estimate	Std. error	t value	P
GRASSES				
Agrostis tenuis				
**Total biomass**<br>glm(formula = Total biomass~ 1, family = Gamma, link = identity)				
Intercept	67.005	6.889	9.727	$2.2e^{-13}$
Deviance Null/ Residual 41.337 (df =53)				
**Shoot/root ratio**<br>glm(formula = shoot/root ratio~1, family = Gamma, link = identity)				
Intercept	0.84779	0.06862	12.35	$<2e^{-16}$
Deviance Null/ Residual 18.172 (df =53)				
Anthoxanthum odoratum				
**Total biomass**<br>glm(formula = Total biomass~1, family = Gamma, link = identity)				
Intercept	50.496	4.801	10.52	$1.39e^{-14}$
Deviance Null/ Residual 27.755 (df =53)				
**Shoot/root ratio***<br>glm(formula = shoot/root ratio ~ Light treatment, family = Gamma, link = identity)				
Intercept	1.4260	0.2012	7.088	$4.37e^{-09}$
L2	0.5113	0.3458	1.479	0.146
L3	-0.1110	0.2737	-0.406	0.687
Deviance Null 18.335 (df =52) , Residual 16.840 (df =50)				
Dactylis glomerata				
**Total biomass**<br>glm(formula = Total biomass ~ Light treatment + Trophic treatment, family = Gamma, link = identity)				
Intercept	35.017	6.276	5.580	$1.09e^{-06}$
L2	14.541	7.888	1.843	0.0714
L3	15.639	8.002	1.954	0.0565
T2	14.360	9.033	1.590	0.1185
T3	-5.053	7.314	-0.691	0.4930
Deviance Null 15.389 (df =52) , Residual 13.149 (df =48)				
**Shoot/root ratio**<br>glm(formula = shoot/root ratio ~ Light treatment, family = Gamma, link = identity)				
Intercept	1.7476	0.2380	7.341	$1.76e^{-09}$
L2	-0.4876	0.2907	-1.677	0.0997
L3	-0.3513	0.3014	-1.166	0.2493
Deviance Null 12.260 (df =52) , Residual 11.269 (df =50)				
Festuca ovina				
**Total biomass**<br>glm(formula = Total biomass ~ 1, family=Gamma, link = identity)				
Intercept	5.445	1.286	4.234	$1e^{-04}$
Deviance Null/ Residual 96.157 (df = 49)				
**Shoot/root ratio**<br>glm(formula = shoot/root ratio ~ Light treatment * Trophic treatment ,family=Gamma, link = identity)				
Intercept	1.394926	0.417320	3.343	0.00178
L2	-0.522842	0.525605	-0.995	0.32570
L3	0.849654	0.790623	1.075	0.28881

T2	-0.697695	0.466547	-1.495	0.14246
T3	-0.007179	0.588663	-0.012	0.99033
L2:T2	1.123107	0.738775	1.520	0.13613
L3:T2	-0.109036	0.923923	-0.118	0.90663
L2:T3	0.984184	0.868707	1.133	0.26383
L3:T3	-1.619029	0.911963	-1.775	0.08327
Deviance Null 30.357 (df = 49) , Residual 22.159 (df = 41)				
Holcus lanatus				
**Total biomass** glm(formula = Total biomass ~ Light treatment + Trophic treatment, family = Gamma, link = identity)				
Intercept	29.541	8.065	3.663	0.000642
L2	-5.380	4.393	-1.225	0.226906
L3	-5.419	4.350	-1.246	0.219216
T2	-17.324	7.824	-2.214	0.031810
T3	-19.326	7.674	-2.518	0.015335
Deviance Null 80.226 (df = 50) , Residual 58.885 (df = 46)				
**Shoot/root ratio** glm(formula = shoot/root ratio ~ Light treatment * Trophic treatment, family=Gamma, link = identity)				
Intercept	1.41266	0.26532	5.324	$3.69e^{-06}$
L2	-0.38675	0.33904	-1.141	0.2605
L3	0.08459	0.38662	0.219	0.8279
T2	1.02738	0.52955	1.940	0.0591
T3	0.15444	0.41755	0.370	0.7133
L2:T2	0.27112	0.74410	0.364	0.7174
L3:T2	-1.38879	0.63640	-2.182	0.0347
L2:T3	0.63794	0.57925	1.101	0.2770
L3:T3	-0.75386	0.53091	-1.420	0.1630
Deviance Null 13.7577 (df = 50) , Residual 8.3805 (df = 42)				
HERBS				
Lotus corniculatus				
**Total biomass** glm(formula = Total biomass ~ Light treatment + Trophic treatment, family=Gamma, link = identity)				
Intercept	37.4952	10.7500	3.488	0.00116
L2	3.4325	4.9427	0.694	0.49122
L3	0.1926	3.7705	0.051	0.95950
T2	-30.7734	10.7118	-2.873	0.00635
T3	-25.0518	11.0481	-2.268	0.02856
Deviance Null 67.88 (df = 46) , Residual 46.53 (df = 42)				
**Shoot/root ratio*** glm(formula = shoot/root ratio~1, family = Gamma, link = identity)				
Intercept	0.47767	0.05913	8.079	$2.65e^{-10}$
Deviance Null/Residual 30.129 (df = 45)				
Achillea millefolium				
**Total biomass** glm(formula = Total biomass ~ Light treatment, family = Gamma, link = identity)				
Intercept	15.569	6.129	2.540	0.0147
L2	8.940	11.699	0.764	0.4488

L3	4.015	9.849	0.408	0.6855
Deviance Null 67.880 (df = 46) , Residual 66.289 (df = 44)				
**Shoot/root ratio** glm(formula = shoot/root ratio ~ 1, family = Gamma, link = identity)				
Intercept	0.69584	0.07241	9.61	$4.03e^{-13}$
Deviance Null/Residual 18.338 (df = 52)				
Centaurea nigra				
**Total biomass** glm(formula = Total biomass ~ Light treatment +Trophic treatment, family = Gamma, link = log)				
Intercept	1.5796	0.2421	6.525	$3.61e^{-08}$
L2	0.1216	0.2652	0.459	0.6485
L3	-0.3490	0.2652	-1.316	0.1943
T2	0.5813	0.2652	2.192	0.0331
T3	0.4966	0.2652	1.873	0.0671
Deviance Null 47.015 (df = 53) , Residual 40.935 (df = 49)				
**Shoot/root ratio** glm(formula = shoot/root ratio ~ 1, family = Gamma, link = identity)				
Intercept	0.6676	0.0712	9.377	$7.6e^{-13}$
Deviance Null/ Residual 21.39 (df = 53)				
Hypochaeris radicata				
**Total biomass** glm(formula = Total biomass ~ 1, family = Gamma, link = identity)				
Intercept	47.746	4.222	11.31	$9.63e^{-16}$
Deviance Null/ Residual 25.559 (df = 53)				
**Shoot/root ratio*** glm(formula = shoot/root ratio ~ Light treatment * Trophic treatment, family = Gamma, link = identity)				
Intercept	1.1340	0.1966	5.769	$7.37e^{-07}$
L2	-0.3376	0.2402	-1.405	0.167
L3	0.1221	0.2934	0.416	0.679
T2	0.3671	0.3261	1.126	0.266
T3	0.3173	0.3385	0.937	0.354
L2:T2	0.5734	0.4648	1.234	0.224
L3:T2	-0.5756	0.4321	-1.332	0.190
L2:T3	-0.1406	0.4026	-0.349	0.729
L3:T3	-0.5502	0.4398	-1.251	0.218
Deviance Null 10.9054 (df = 52) , Residual 8.0476 (df = 44)				
Lotus pedunculatus				
**Total biomass** glm(formula = Total biomass ~ Light treatment, family = Gamma, link = identity)				
Intercept	72.888	9.199	7.923	$1.92e^{-10}$
L2	-16.492	11.632	-1.418	0.1623
L3	-25.949	10.942	-2.371	0.0215
Deviance Null 22.059 (df = 53) , Residual 20.286 (df = 51)				
**Shoot/root ratio*** glm(formula = shoot/root ratio ~ 1, family = Gamma, link = identity)				
Intercept	0.62941	0.05249	11.99	$<2e^{-16}$
Deviance Null/ Residual 22.547 (df = 52)				

Plantago lanceolata				
**Total biomass**<br>glm(formula = Total biomass ~ 1, family = Gamma, link = identity)				
Intercept	8.2500	0.8125	10.15	$7.65e^{-14}$
Deviance Null/ Residual 21.008 (df = 51)				
**Shoot/root ratio**<br>glm(formula = shoot/root ratio ~ 1, family = gaussian)				
Intercept	0.96122	0.06096	15.77	$<2e^{-16}$
Deviance Null/ Residual 9.8556 (df = 51)				
Prunella vulgaris				
**Total biomass**<br>glm(formula = Total biomass ~ Light treatment + Trophic treatment, family = Gamma, link = identity)				
Intercept	0.5980	0.2019	2.962	0.00563
L2	-0.2984	0.2993	-0.997	0.32607
L3	-0.4383	0.2060	-2.127	0.04099
T2	0.3428	0.2078	1.649	0.10859
T3	0.4358	0.2174	2.004	0.05332
Deviance Null 39.242 (df = 37) , Residual 32.284 (df = 33)				
**Shoot/root ratio***<br>glm(formula = shoot/root ratio ~ Light treatment +Trophic treatment, family = gaussian)				
Intercept	2.3099	0.3499	6.602	$1.92e^{-07}$
L2	0.4825	0.4195	1.150	0.25854
L3	0.5498	0.3470	1.584	0.12294
T2	-1.3426	0.4244	-3.163	0.00341
T3	-0.5875	0.4180	-1.406	0.16948
Deviance Null 38.362 (df = 36) , Residual 27.000 (df = 32)				
Trifolium pratense				
**Total biomass**<br>glm(formula = Total biomass ~ Light treatment + Trophic treatment, family = Gamma, link = identity)				
Intercept	19.6743	3.6187	5.437	$2e^{-06}$
L2	-0.4816	2.9830	-0.161	0.8725
L3	-1.8968	2.8216	-0.672	0.5048
T2	-8.2036	3.6305	-2.260	0.0286
T3	-7.9997	3.6712	-2.179	0.0345
Deviance Null 24.686 (df = 50) , Residual 20.982 (df = 46)				
**Shoot/root ratio**<br>glm(formula = shoot/root ratio ~ Light treatment +Trophic treatment, family = Gamma, link = identity)				
Intercept	2.43302	0.43328	5.615	$1.09e^{-06}$
L2	0.01443	0.44310	0.033	0.974
L3	-0.42500	0.39651	-1.072	0.289
T2	0.26422	0.51499	0.513	0.610
T3	-0.87290	0.41290	-2.114	0.040
Deviance Null 21.936 (df = 50) , Residual 18.669 (df = 46)				

Table S4. Analysis of deviance for GLM fits. Leaf nitrogen content (%) and leaf C:N ratio per species (N = 10) were modelled as a function of light treatment (L1 = unlit control, L2 = amber light (simulating LPS lighting), and L3 = white LED lighting), and trophic treatment (T1 = plants only, T2= plants and herbivores, and T3= plants, herbivores, and predators.). Significance tested against its intercept only model (null model). df = degrees of freedom. Model and error structures are specified for each model selected. Coefficients relative to the base level of each factor (light treatment = unlit controls; trophic treatment = plants only).

Fixed effects	Estimate	Std. error	t value	P
GRASSES				
Agrostis tenuis				
**Leaf nitrogen content**<br>glm(formula = Nitrogen ~ 1, family=gaussian)				
Intercept	1.65280	0.04526	36.52	$<2e^{-16}$
Deviance Null/ Residual 5.0188 (df = 49)				
**leaf C:N ratio**<br>glm(formula = C:N ratio ~1, family = Gamma, link = identity)				
Intercept	32.0566	0.7711	41.57	$<2e^{-16}$
Deviance Null/ Residual 1.4692 (df = 49)				
Anthoxanthum odoratum				
**Leaf nitrogen content**<br>glm(formula = Nitrogen ~ 1, family = Gamma, link = identity)				
Intercept	1.47882	0.05256	28.14	$<2e^{-16}$
Deviance Null/Residual 3.023 (df =50)				
**leaf C:N ratio**<br>glm(formula = C:N ratio ~1, family = Gamma, link = identity)				
Intercept	35.715	1.192	29.97	$<2e^{-16}$
Deviance Null/ Residual 2.9885 (df = 50)				
Dactylis glomerata				
**Leaf nitrogen content**<br>glm(formula = Nitrogen ~ 1, family = gaussian)				
Intercept	1.76021	0.05861	30.03	$<2e^{-16}$
Deviance Null/ Residual 7.7501 (df = 47)				
**leaf C:N ratio**<br>glm(formula = C:N ratio ~1, family = Gamma, link = identity)				
Intercept	30.262	1.063	28.48	$<2e^{-16}$
Deviance Null/ Residual 2.5643 (df = 47)				
Holcus lanatus				
**Leaf nitrogen content**<br>glm(formula = Nitrogen ~ 1, family = Gamma, link = identity)				
Intercept	1.77697	0.06871	25.86	$<2e^{-16}$
Deviance Null/ Residual 1.8215 (df = 35)				
**leaf C:N ratio**<br>glm(formula = C:N ratio ~1, family = Gamma, link = identity)				
Intercept	29.02	1.06	27.37	$<2e^{-16}$
Deviance Null/ Residual 1.7365 (df = 35)				
HERBS				
Lotus corniculatus				
**Leaf nitrogen content**<br>glm(formula = Nitrogen ~ Light treatment * Trophic treatment, family = Gamma, link = identity)				
Intercept	2.89500	0.21434	13.507	$9.14e^{-15}$
L2	0.33700	0.30290	1.113	0.2742
L3	-0.06300	0.28480	-0.221	0.8263
T2	-0.05250	0.30039	-0.175	0.8624
T3	0.04900	0.28974	0.169	0.8668
L2:T2	-0.72950	0.42416	-1.720	0.0951
L3:T2	1.08383	0.48424	2.238	0.0323

L2:T3	0.05733	0.41290	0.139	0.8904
L3:T3	0.01567	0.38702	0.040	0.9680
Deviance Null 1.18425 (df = 40), Residual 0.72352 (df = 32)				
**leaf C:N ratio**<br>glm(formula = C:N ratio ~ Light treatment * Trophic treatment, family = Gamma, link = identity)				
Intercept	17.7225	1.1827	14.985	$5.11e^{-16}$
L2	-1.3945	1.5325	-0.910	0.3696
L3	0.5015	1.6068	0.312	0.7570
T2	-0.1350	1.6662	-0.081	0.9359
T3	-0.0965	1.5829	-0.061	0.9518
L2:T2	4.7937	2.5182	1.904	0.0660
L3:T2	-4.5823	2.2456	-2.041	0.0496
L2:T3	-0.6282	2.0440	-0.307	0.7606
L3:T3	-0.4258	2.1492	-0.198	0.8442
Deviance Null 0.96131 (df = 40), Residual 0.57313 (df = 32)				
Centaurea nigra				
**Leaf nitrogen content**<br>glm(formula = Nitrogen~1, family = gaussian)				
Intercept	1.80704	0.05901	30.62	$<2e^{-16}$
Deviance Null/ Residual 8.5309 (df = 49)				
**leaf C:N ratio**<br>glm(formula = C:N ratio ~1, family = Gamma, link = identity)				
Intercept	28.4903	0.8166	34.89	$<2e^{-16}$
Deviance Null/ Residual 2.0786 (df = 49)				
Hypochaeris radicata				
**Leaf nitrogen content**<br>glm(formula = Nitrogen ~ 1, family = Gamma, link = identity)				
Intercept	1.99457	0.08016	24.88	$<2e^{-16}$
Deviance Null/Residual 4.2631 (df = 52)				
**leaf C:N ratio**<br>glm(formula = C:N ratio ~1, family = Gamma, link = identity)				
Intercept	25.4345	0.9954	25.55	$<2e^{-16}$
Deviance Null/ Residual 4.0389 (df = 52)				
Lotus pedunculatus				
**Leaf nitrogen content**<br>glm(formula = Nitrogen ~ Light treatment + Trophic treatment, family = Gamma, link = identity)				
Intercept	3.7772	0.2336	16.173	$< 2e^{-16}$
L2	-0.2272	0.2293	-0.991	0.32696
L3	-0.2094	0.2374	-0.882	0.38231
T2	-0.6910	0.2321	-2.977	0.00463
T3	-0.2533	0.2464	-1.028	0.30933
Deviance Null 2.2629 (df = 50), Residual 1.8390 (df = 46)				
**leaf C:N ratio**<br>glm(formula = C:N ratio ~ Light treatment + Trophic treatment, family = Gamma, link = identity)				
Intercept	14.5394	0.9548	15.228	$< 2e^{-16}$
L2	0.7857	1.0634	0.739	0.46377
L3	0.7744	1.0955	0.707	0.48320
T2	3.1987	1.1209	2.854	0.00646
T3	0.7340	1.0339	0.710	0.48134
Deviance Null 2.1602 (df = 50), Residual 1.7842 (df = 46)				

Plantago lanceolata				
**Leaf nitrogen content**<br>glm(formula = Nitrogen ~ 1, family = Gamma, link = identity)				
Intercept	1.36574	0.08042	16.98	$<2e^{-16}$
Deviance Null/Residual 6.7 (df = 46)				
**leaf C:N ratio**<br>glm(formula = C:N ratio ~ 1, family = Gamma, link = identity)				
Intercept	41.778	2.163	19.31	$<2e^{-16}$
Deviance Null/ Residual 6.0536 (df = 46)				
Trifolium pratense				
**Leaf nitrogen content**<br>glm(formula = Nitrogen ~ 1, family=gaussian)				
Intercept	3.10400	0.08291	37.44	$<2e^{-16}$
Deviance Null/ Residual 13.61 (df = 44)				
**leaf C:N ratio**<br>glm(formula = C:N ratio ~ 1, family = Gamma, link = identity)				
Intercept	17.0822	0.3607	47.35	$<2e^{-16}$
Deviance Null/ Residual 0.91195 (df = 44)				

TABLE S5. Analysis of deviance for GLM fits. Plant species richness per mesocosm was modelled as a function of the light treatment (L1 = unlit control, L2 = amber light (simulating LPS lighting), and L3 = white LED lighting). Significance tested against its intercept only model (null model). df = degrees of freedom. Model and error structures are specified. Coefficients relative to the base level of the factor (unlit controls).

**Fixed effects**	**Estimate**	**Std. error**	**z value**	**P**
**Plant species richness per mesocosm**				
glm(formula = Number of species ~ Light treatment, family = poisson)				
Intercept	2.63109	0.06325	41.601	$<2e^{-16}$
L2	-0.06134	0.09373	-0.654	0.513
L3	-0.01005	0.08739	-0.115	0.908
Deviance Null 7.6416 (df = 53) , Residual 7.1547 (df = 51)				
